# Electrospun Nanofibers for Chemical Separation

**DOI:** 10.3390/nano10050982

**Published:** 2020-05-21

**Authors:** Mesbah Najafi, Margaret W. Frey

**Affiliations:** Department of Fiber Science & Apparel Design, Cornell University, Ithaca, NY 14853, USA; mfw24@cornell.edu

**Keywords:** polymer, membrane, electrospinning, nanofiber, separation, adsorption

## Abstract

The separation and purification of specific chemicals from a mixture have become necessities for many environments, including agriculture, food science, and pharmaceutical and biomedical industries. Electrospun nanofiber membranes are promising materials for the separation of various species such as particles, biomolecules, dyes, and metals from liquids because of the combined properties of a large specific surface, light weight, high porosity, good connectivity, and tunable wettability. This paper reviews the recent progress in the design and fabrication of electrospun nanofibers for chemical separation. Different capture mechanisms including electrostatic, affinity, covalent bonding, chelation, and magnetic adsorption are explained and their distinct characteristics are highlighted. Finally, the challenges and future aspects of nanofibers for membrane applications are discussed.

## 1. Introduction

In recent years, membrane technologies have obtained great popularity for chemical separations based on their high separation efficiency, simple operational process, and relatively low production cost. A membrane acts like a barrier between two phases and allows a substance to be selectively transferred from one side to the other [1]. It can capture various types of species such as particles, proteins, cells, antibodies, metal ions, and dyes based on their special characteristics (e.g., charge, affinity, size). Membranes have a considerable role in various environmental, agricultural, pharmaceutical, and biomedical applications. They are utilized inside filter devices to purify water, liquids, solutions, and blood for water treatment, artificial kidneys, and control drug delivery applications. They have also been used inside small analytical devices such as biosensors for the in situ analysis of pollutants in crops and soils, the detection and identification of infectious diseases in crops and livestock, and the screening of therapeutic drugs in veterinary testing [2,3,4,5,6].

Nanofiber membranes with a superb surface area to volume ratio, high porosity/tortuosity, and high permeability are an excellent choice for the fabrication of membranes. Several techniques such as nanolithography, phase separation, self-assembly, electrospinning, melt-blowing, and template synthesis have been used for the production of nanofibers. Among them, electrospinning is of particular interest because of its simplicity, versatility, flexibility, and the ease of processing [7]. This technique only requires a syringe, a flat tip needle, a high voltage power supply, and a conducting collector. In terms of flexibility, electrospinning is able to fabricate continuous nanofibers from a wide range of materials such as polymers, composites, semiconductors, and ceramics [7,8,9].

**Figure 1:**
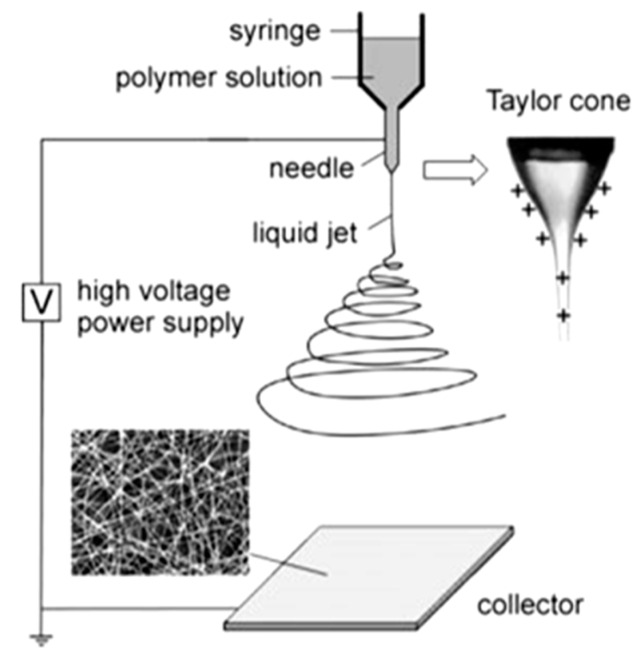
Scheme of the electrospinning process (Reproduced with permission from [10]. Copyright Wiley, 2004).

Electrospinning is a fiber formation method in which an electrical field is applied to polymer solutions or polymer melts to produce micro- and nanofibers. A typical electrospinning process comprises three main components: a feeding section, a high voltage power supply, and a collector section (Figure 1). The feeding part comprises a feed storage unit (polymer solution/melt), a spinneret (a thin metallic needle), and a syringe pump to inject the solution/melt at a constant flow/feed rate. The voltage device is used to create an electrically charged polymer solution/melt out of the spinneret [7]. The collector involves a counter or grounded metal electrode attached to a rotating drum or a flat plate. When the electrical feed increases over a critical value, the electrical force overcomes the solution surface tension, and this changes the hemispherical shape of the solution drop at the needle tip into a conical shape known as a Taylor cone, which discharges/ejects a solution jet onto the collector screen. The jet elongates under the electrical field while the solvent evaporates, leaving behind solidified polymer fibers as an interconnected web on the collector [7].

**Figure 2:**
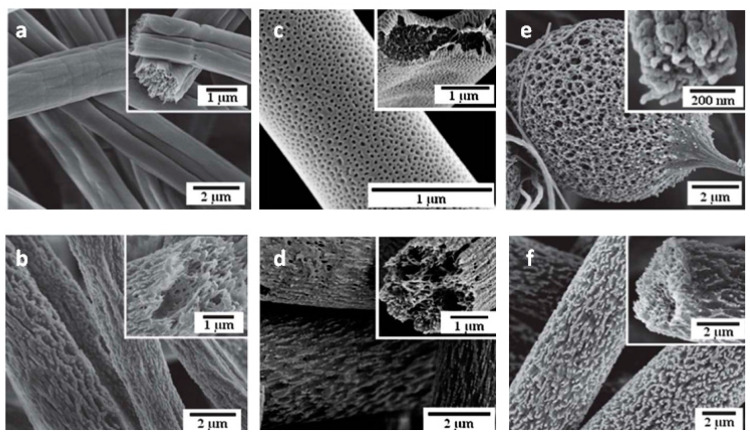
SEM images of electrospun polystyrene (PS) nanofibers produced at two molecular weights of (**a**) M_w_ = 34 KD and (**b**) M_w_ = 208 KD [13], morphology of PS nanofibers (M_w_ = 208 KD) formed from tetrahydrofuran (THF) and dimethylformamide (DMF) solvents: (**c**) THF/DMF (100/0) (**d**) THF/DMF (40/60), PS (*M_w_* = 208 KD) fibers nanofibers electrospun from (**e**) 10% and (**f**) 30% PS in THF:DMF (80/20 *w/w*) [13] (Reproduced with permission from [13]. Copyright Elsevier, 2012).

The effect of the electrospinning conditions on the morphological and structural characteristics of the nanofibers have been discussed in the literature [7,8,11]. The nanofibers’ diameter, shape, and surface topography depend on several solution/melt properties such as viscosity, molecular weight, molecular weight distribution, pH value, polymer conformations, electrical conductivity, surface tension, and the solvent evaporation rate, as well as the processing/spinning conditions, including the geometry and size of the collector mandrel, feed rate, working distance, and strength of the electrical field, and ambient parameters such as temperature, air speed, and humidity [12,13,14]. Manipulating these operating/material conditions can result in different fiber morphologies such as beaded, porous, branched, surface, inner, and hollow structures (Figure 2). Ideal nanofibrous fabrics should have a continuous single nanofiber with a defect-free surface structure and constant and controllable diameters [8].

Nanofiber membranes require several properties to be used for chemical separation. Depending on the application, they need to have a high porosity, appropriate pore size, as well as a high mechanical, chemical, and/or thermal resistance, as they may experience different temperatures, pH, stresses, and vibrations in a liquid flow. In addition, a nanofibrous membrane often needs to have a good selectivity and reusability to increase the separation performance and operational flexibility, and to reduce the material and labor costs. Nanofibrous fabrics can separate chemicals and impurities by different capture mechanisms including electrostatic, affinity, covalent bonding, chelation, magnetic, and size exclusion/filtration. The application of electrospun membranes for liquid filtration and oil/water separation have been reviewed by other authors [11,12]. As such, in this contribution, we will focus on the recent development of electrospun nanofibers working with other capture mechanisms.

## 2. Nanofiber Adsorption Mechanisms

### 2.1. Ion Exchange

Ion exchange membranes (IEMs) have been widely used for the separation/removal of dissolved ions from a liquid solution [15]. Electrospinning is a promising method for the fabrication of IEMs as the resultant membranes have a large specific surface area and an interconnected porous structure. The capture mechanism of these membranes is based on the electrostatic attraction between the charged membrane and the counter ions. IEMs can have positively charged groups (e.g., –NH_3_^+^, –NRH_2_^+^, –NR_2_H^+^, –NR_3_^+^, –PR_3_^+^, –SR_2_^+^) or negatively charged groups (e.g., –SO_3_^−^, –COO^−^, –PO_3_^2−^, –PO_3_H^−^, –C_6_H_4_O^−^) in their chemical structures [16]. These functional groups can be either in the primary composition of polymer membranes, or can be introduced into the membrane structure by utilizing charged polymers/particles and surface modification methods.

Electrospun IEMs have been used for the selective separation of a variety of molecules such as proteins, cells, antibodies, and metal ions. By regulating the pH, these chemicals obtain negative/positive charges and can be adsorbed on oppositely charged nanofibers. Ki et al. [17] fabricated electrospun membranes from wool keratose (WK) and silk fibroin (SF) proteins for the adsorption of heavy metal ions. To achieve that, they blended WK and SF to improve the spinnability and the structural stability of WK in water. The membrane exhibited an adsorption capacity of 2.88 μg/mg at pH 7 towards copper ions thanks to the high amount of amino acids in the silk/wool structure. In another study, Horzum et al. [18] examined the adsorption performance of elecrospun chitosan nanofibers towards different metal ions. They reported a high sorption capacity of 600 mg/g at pH 6 due to the combined electrostatic and chelating mechanisms between the positively charged amines and the negatively charged metal ions. 

**Figure 3:**
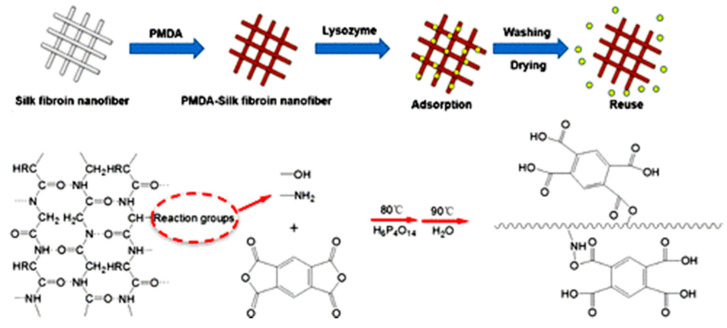
Graft reactions between silk and PMDA for lysozyme adsorption (adapted from [22] with the permission of American Chemical Society).

If a membrane does not have an appropriate functional group for chemical adsorption, its surface chemistry needs to be modified. Grafting is one of the most common methods for enhancing the surface functionality of electrospun nanofibers. Wang et al. [19] combined electrospinning with in situ graft polymerization of PVA and maleic anhydride (MAH) to make nanofibers for protein separation. The resultant PVA/MAH membranes exhibited an adsorption capacity of 177 mg/g for lysozyme at pH 6. In addition, Fu et al. [20] grafted citric acid (CCA) to electrospun ethylene-vinyl alcohol (EVOH) nanofibers for protein adsorption. The obtained EVOH−CCA membranes showed a high static adsorption capacity of 284 mg/g toward lysozyme at pH 6 thanks to a large amount of carboxyl groups of CCA on the nanofiber surface. Moreover, Zhu et al. [21] functionalized the surface of electrospun PVA/PEI with a thin coating of polydopamine/polyethyleneimine (PDA/PEI). The obtained core–shell structured PDA/PEI@PVA/PEI adsorbed Ponceau S and Methylene Blue dyes with adsorption capacities of 1180 and 1290 mg/g, respectively. In another study, Yi et al. [22] modified the surface chemistry of electrospun silk membranes by grafting pyromellitic dianhydride (PMDA) to the nanofibers. The amino and hydroxyl groups at the side chain of the silk could easily react with PMDA (Figure 3), and generate negatively charged carboxyl groups which captured lysozyme with a robust capacity of 710 mg/g at pH < 7. 

**Figure 4:**
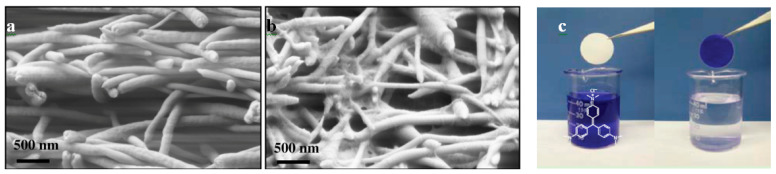
(**a**) SEM images of PAN electrospun nanofibers, (**b**) cellulose nanowhisker-modified PAN electrospun nanofibers and (**c**) adsorption of crystal violet dye by the cellulose nanowhisker-modified PAN nanofibers (Reproduced with permission from [26]. Copyright American Chemical Society, 2012).

The incorporation of nanowhiskers and nanoparticles into nanofibers is another way to improve a membrane’s surface chemistry [23,24,25,26]. Chu and coworkers [26] used ultrafine cellulose whiskers in electrospun polyacrylonitrile/polyethylene terephthalate (PAN/PET) scaffolds. The impregnated cellulose nanowhiskers had a very high negative surface charge density and provided a high adsorption capacity for the removal of positively charged species, such as crystal violet (CV) dye (Figure 4). The capture capacity of the obtained membrane was 16 times higher than that of the equivalent commercial membranes. Furthermore, Fan et al. [25] utilized SiO_2_ nanoparticles in a PAN spinning solution to fabricate carbon nanofibers with robust mechanical properties for protein adsorption. To activate the nanofiber surface with N-H groups, the emitted nitrogen gas from the PAN carbonization process was reused. The resultant SiO_2_@CNF membrane possessed a bovine serum albumin (BSA) adsorption capacity of 30 ± 0.9 mg/g due to the surface roughness of the nanofibers, electrostatic attraction, and hydrophobic adsorption between the carbon and BSA protein.

**Figure 5:**
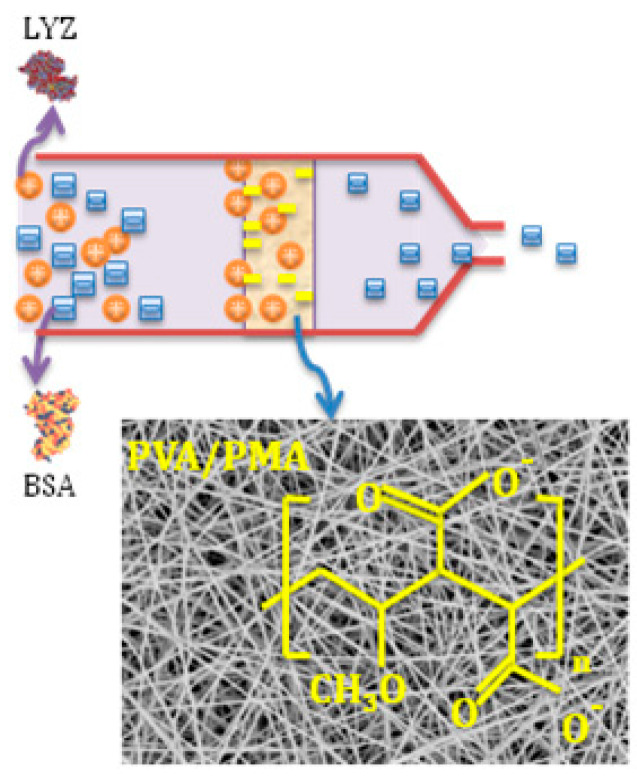
PVA-PMA nanofiber membranes for proteins separation; at pH 6, the charge of LYZ (IP of 10.8) is positive, while the charges of the BSA (IP = 4.8) and PVA/PMA nanofibers are both negative. Thus, the nanofibers repel the BSA and capture the LYZ, resulting in a selective protein adsorption from a mixture.

Moreover, the charged chemical groups can be induced to the nanofiber surface by cationic and anionic polymers. Our research group, previously [27,28,29,30], functionalized the surface of PVA nanofibers by incorporating cationic/anionic polymers such as poly(hexadimethrine bromide) (PB) with amine groups or poly(methyl vinyl ether-alt-maleic anhydride) (PMA) with carboxyl groups. They used these nanofibers for the separation of dyes [27], proteins (Figure 5) [31], liposomes [29], and Escherichia coli (*E. coli)* cells [28] for water purification and medical diagnosis applications. In one study [31], for example, a percentage of PMA was added to PVA, which resulted in composite nanofibers with the combined properties of appropriate adsorbent groups (from PMA) and high mechanical properties (from PVA). A high adsorption capacity of 476.53 ± 19.48 mg/g was obtained at pH 6, owing to the electrostatic attraction between the negatively charged nanofibers and positively charged proteins. Furthermore, Min et al. [32] incorporated the cationic polymer poly-ethyelenimine (PEI) into polyether sulfone (PES) nanofibers (PS/PEI membranes) for anionic dye and metal ion adsorption. They reported superb adsorption capacities of 1000 mg/g (at pH 1) and 357.14 mg/g (at pH 5–7) for Sunset Yellow FCF and Cd(II), respectively. 

In addition, appropriate functional adsorption groups can be introduced into the nanofibers by chemical treatments. Chiu et al. treated electrospun PAN nanofibers with sodium hydroxide (NaOH) to transform the cyanide functional groups (–C≡N) to hydrophilic carboxyl functional groups (–COOH) [33]. They reported a lysozyme adsorption capacity of about 105 mg/g (at pH 9) which was two times higher than that of the available commercial products. Additionally, Schneiderman et al. [34] functionalized the surface of carbon nanofibers with nitric acid at 90 °C for 48 hrs to carboxylate the nanofiber surface for protein adsorption. The capture capacity of the nanofiber mats was approximately 10 times higher than that of their microfiber counterparts. In another study, Li et al. [35] fabricated pH-controllable electrospun nanofibers by functionalizing polyacrylonitrile (PAN) nanofibers with lysine (LYS) for the selective adsorption of proteins. By tailoring the pH, they were able to create positive and negative charges on the nanofiber surface. Maximum adsorption capacities of 425.49 mg/g at pH 3 and 54.98 mg/g at pH 8 were reported for capturing pepsin (Isoelectric point (IP) = 1) and lysozyme (IP = 10.8), respectively.

Plasma treatment can also be used for the surface functionalization of nanofiber-based IEMs. Doraki et al. [36] modified the surface of electrospun chitosan/polyethylene oxide (90/10, *v*/*v*) nanofibers with air dielectric barrier discharge (DBD) plasma for acetylcholinesterase (AChE) enzyme immobilization. They reported that a 6 min plasma treatment induces NH_3_^+^ polar groups on the membrane surface which can immobilize negatively charged AChE (IP ~5.87) at pH 7.4. Although they did not report the enzyme capture capacity of the membrane, the activity of the enzyme immobilized on the plasma-treated membrane was about 12% more than that of the unmodified membrane. Furthermore, Najafi et al. [37] recently applied an oxygen plasma treatment to an electrospun PLA nanofiber mat to introduce carboxylic functional groups to the fabric surface. The obtained nanofibers effectively captured Methylene Blue dye with the electrostatic adsorption mechanism from a dye solution. Plasma treatment is a simple method for improving the surface chemistry of polymer membranes. Yet, as it only affects the surface layer of the nanofibers, the adsorption capacity is less than the other techniques discussed in this section.

**Figure 6:**
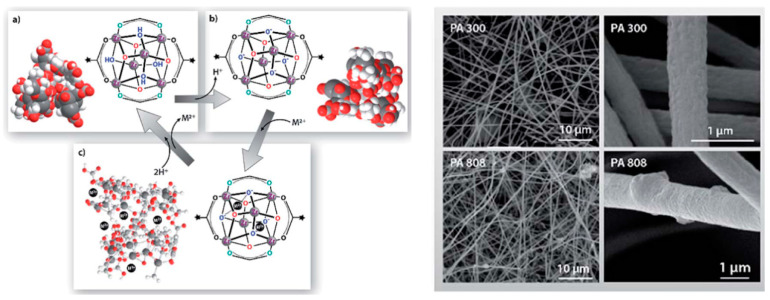
(Left) Electrostatic adsorption between MOF 808 and heavy metals at the surface and pore space caused by a change in pH. (**a**) MOF 808, (**b**) deprotonated MOF 808, and (**c**) heavy metal ion bound MOF. M^2+^ refers to heavy metal ions; black dots represent adsorbed M^2+^. Color code: Zr = grey, C = ash, O = red, H = white. (Right) SEM images of the nanofibrous membranes with 20 wt% MOF loading: PA 300 and PA 808 (Reproduced with permission from [38]. Copyright The Royal Society of Chemistry, 2018).

Metal–organic frameworks (MOF) can also be used for the fabrication of IEMs for liquid purification. MOFs, also known as porous coordination polymers, are multifunctional materials consisting of metal ions or clusters and organic linkers. The extraordinary porosity, tunable pore sizes, and high surface area to mass ratios (>6000 m^2^/g) make these materials excellent adsorbents for the separation of heavy metals [38,39,40]. MOFs have been used with electrospun nanofibers for controlling air pollution. However, because of their low stability in aqueous media, their application for liquid purification has been limited [41]. Efome et al. [38] recently reported on MOF nanofibrous membranes for the adsorption of heavy metals in an aqueous medium. The membrane was fabricated by enmeshing MOFs (Zr_6_O_4_(OH)_4_(COOH)_6_(BTC)_2_ and Fe_3_OF–(H_2_O)_2_(BTC)_2_·*n*H_2_O (MOF 808 and F300)) based on iron (III) and zirconium (IV) in polyacrylonitrile (PAN) and polyvinylidene fluoride (PVDF) electrospun nanofibers (Figure 6). Because of the high compatibility of MOFs and nanofibers, the MOFs did not permeate into the liquid medium even after four cycles of filtration and more than 90% of the adsorption capacity of the membrane was retained. High Hg adsorption capacities of 299.66 and 276.96 mg/g were reported for the MOFs based on iron (III) and zirconium (IV), respectively. The adsorption mechanism was attributed to the electrostatic interaction between the negative charge of the ionized carboxyl group (COO^−^) of the MOF at pH > 5 and the positive charge of the heavy metals [38].

### 2.2. Covalent Attachment

**Figure 7:**
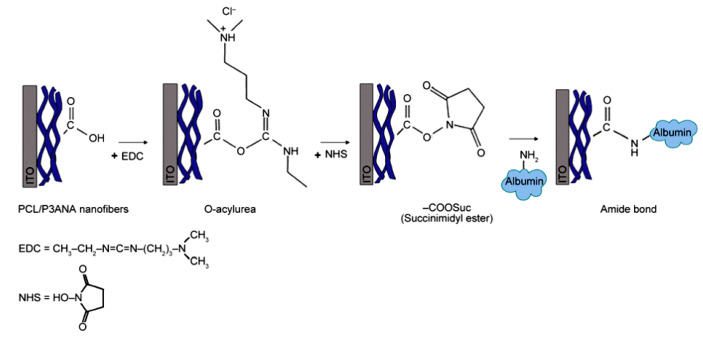
Schematic representation of the covalent attachment of albumin protein on electrospun nanofibers through EDC/NHS activation. EDC/NHS enables proteins to be easily conjugated to a substrate with carboxyl or amino groups (Reproduced with permission from [45]. Copyright Express Polymer Letters, 2016.

Covalent attachment is based on the binding of amino acid residues (–NH_2_, –CO_2_, –SH) of proteins/enzymes to a nanofiber surface [42]. The immobilization enhances many enzyme properties such as heat stability, pH tolerance, functional stability, and performance in organic solvents [43]. Electrospun nanofibers are a good candidate for protein immobilization because of many attractive features including tunable morphology and pore size, as well as compositional variance. Pristine polymer nanofibers often do not have appropriate functional groups for the direct immobilization of enzymes, and they need to be activated by activation agents such as 1-Ethyl-3-(3-dimethylaminopropyl) carbodiimide (EDC), N-Hydroxysuccinimide (NHS), glutaraldehyde (GA), carbonyldiimidazol (CDI), and nitrophenyl chloroformate (NP) [44,45] (Figure 7).

There are several studies regarding the application of polymer nanofibers for protein immobilization. For example, Jia et al. [46] examined the covalent attachment of enzymes onto electrospun polystyrene (PS) nanofibers. In their study, PS was first synthesized with an initiator containing hydroxyl groups. Then, the polymer was functionalized with nitrophenyl (NPh) ending groups, and the product was electrospun into nanofibers. Enzyme α-chymotrypsin (CT) was covalently immobilized onto the nanofiber surface. The amount of enzyme loading was up to 1.4% of the nanofibers, and its specific activity in aqueous solutions was more than 65% of that of the native enzyme. In another study, Lee et al. [47] examined the immobilization of CT enzyme on electrospun silk fibroin nanofibers. They found interesting results on the stability of enzymes on the nanofibers with different diameters. Enzymes on the nanofibers with a 205 nm diameter retained more than 90% of their initial activity in an aqueous solution at 25 °C after 24 h, whereas those loaded on the 320 nm nanofibers showed higher stability in ethanol, retaining more than 45% of their initial activity. A larger fiber diameter decreases the surface area for protein immobilization, resulting in lower enzyme activity.

In these studies, enzymes were successfully attached to the nanofiber surface; however, the captured enzymes only formed a monolayer which limited the enzyme loading on the membrane surface. To address this issue, Gu et al. [48] used a novel and simple approach for enzyme aggregate coatings on nanofibers. In that study, seed enzymes (α-chymotrypsin) were first covalently attached onto electospun poly(styrene-co-maleic anhydride) nanofibers. Then, a glutaraldehyde (GA) treatment was applied for cross-linking and the aggregation/adsorption of additional enzyme molecules from a solution on the seed enzymes. They reported that the initial activity of α-chymotrypsin aggregate-coated nanofibers was nine times higher than that of the nanofibers with only one layer of covalently immobilized α-chymotrypsin molecules. Furthermore, the new aggregation method considerably increased the enzyme stability of α-chymotrypsin aggregate-coated nanofibers with no loss of activity even after over a month of incubation under rigorous shaking conditions. In a similar study, Lee et al. [49] employed this approach to fabricate a β-Glucosidase (βG) enzyme coating on polymer nanofibers. Electrospun nanofibers were produced from a mixture of polystyrene (PS) and polystyrene-co-maleic anhydride (PSMA), and the maleic anhydride groups of PSMA were used for the covalent attachment of enzyme molecules. Their results showed that the apparent initial activity of βG-coated nanofibers by GA cross-linking was 36 times higher than that of the nanofibers with covalently attached βG molecules. Additionally, the βG-coated nanofibers retained 91% of their initial activity after 20 days of incubation under shaking conditions.

The main challenge in immobilizing enzymes on nanofibers is to maintain their stability/activity, as any changes in the reaction conditions may result in the deformation of their structure and the loss of their activity. To address this challenge, the surface chemistry of nanofibers can be modified towards biocompatibility, by utilizing biocompatible polymers in nanofiber electrospinning. Huang et al. [50] tethered collagen on poly(acrylonitrile-co-acrylic acid) (PANCAA) nanofibrous membranes in the presence of EDC/NHS for lipase immobilization. They reported that the activity retention of the immobilized enzyme on the collagen-modified nanofibers was enhanced by up to 61.7%. Additionally, Wang et al. [51] examined the effect of the addition of hydrophilic and biocompatible poly(N-vinyl-2-pyrrolidone) PVP or polyethylene glycol (PEG) polymers to polysulfone electrospun nanofibers on the immobilization of *Candida rugosa* lipases. They reported that the activity of the lipase adsorbed on the composite nanofibers increased with PVP or PEG content, although the lipase adsorption capacity was decreased due to increased fiber diameter and weakened adsorption strength, which was caused by fiber surface hydrophilicity. Another way to improve the retention of enzymes is to use spacer arms on the nanofiber surface. This can offer the enzyme more freedom to move and reduce the steric hindrance induced by the substrate. Wang and Hsieh [52] introduced hydrophilic PEG spacers on the electrospun cellulose nanofibers for lipase immobilization. They found that the fiber-bound lipase exhibited significantly higher catalytic activity in non-polar solvents and at a high temperature. 

### 2.3. Chelation

The chelation/complexation mechanism is based on the formation of two or more separate coordinate bonds between polydentate ligands on a fiber surface and a single central metal ion. Various functional groups such as amino, carboxyl, phosphoric, imidazoline, thioamido, and amidoxime have a complexing ability towards chemical/dissolved ions [53]. These chelating sites can be inside the principle structure of polymer nanofibers or they can be introduced into the membrane by chemical treatments. The adsorption capacity depends on the strength and the number of complexes formed between the metal ions and the adsorbents.

Several researchers have used the chelation mechanism for capturing chemicals on nanofibers. For example, Haider and Park [54] examined the metal adsorbability of electrospun chitosan nanofibers in an aqueous solution. They reported high capture capacities of 485.44 and 263.15 mg/g for Cu(II) and Pb(II), respectively, which were about 6 and 11 times higher than those of the chitosan microsphere and the plain chitosan, respectively. Such superb adsorption capacities were due to the large specific surface area resulting from the small fiber diameter (~235 nm) and the porous structure, as well as the large number of chelating groups (–NH_2_) of chitosan. Moreover, Xiao et al. [55] examined the capability of electrospun polyacrylic acid/polyevinyl alcohol (PAA/PVA) mats for the removal of copper (II) ions in aqueous solutions. The obtained composite mat removed up to 91% of the copper ions due to the strong complexation between the available carboxyl groups of PAA and the copper ions. In another study, Wang et al. [56] produced cross-linked electrospun polyethylenimine (PEI) nanofibrous membranes doped with PVA by a wet-electrospinning process for the adsorption of heavy metal ions. To increase the water stability of the nanofibers, the polymer chains were cross-linked by using a coagulation bath containing glutaraldehyde (GA) during the spinning process. The chelation between the metal ions and the nitrogen atoms of PEI resulted in maximum adsorption capacities of 70.92, 121.95, and 94.34 mg/g for Cu(II), Cd(II), and Pb(II), respectively. Moreover, Wu et al. [57] produced thiol-functionalized PVA/SiO_2_ composite nanofibers via sol-gel electrospinning. A viscous PVA/gel gel was obtained by adding a percentage of PVA solution to a silica gel containing 3-Mercaptopropyltrimethoxysilane (MPTMS, 99%). They reported a large adsorption capacity of 489.12 mg/g for heavy metal ions thanks to the nanofibers’ large surface area of 290 m^2^/g. Such adsorption was attributed to the electrostatic attraction and complex formation between the metal ions and the thiol groups. 

If a membrane does not have a chelating group, its surface chemistry can be modified by chemical treatment methods. Kampalanonwat and Supaphol [58] modified/hydrolyzed the surface of PAN nanofibers with a sodium hydroxide ethanolic/aqueous solution to transform the nitrile groups of PAN into imine conjugate sequences (–C=N–). A capture capacity of 31.3 mg/g was reported for the Cu(II) ions, and the adsorbed metals could be fully recovered upon submersion in a 0.1 M aqueous HCl solution for 30 min. In addition, Saeed et al. [53] prepared amidoxime PAN (PAN–oxime) nanofibers by combining the electrospinning technique and the chemical modification of the nitrile group in PAN. The oxime group (C=N–OH) was introduced on the fiber surface via the reaction of hydroxylamine hydrochloride (NH_2_OH) and sodium carbonate (Na_2_CO_3_) with the PAN nanofiber nitrile group. Adsorption capacities of 52.70 and 263.45 mg/g were reported for Cu(II) and Pb (II), respectively, due to the chelation between the available oxime sites on the fibers and the heavy metal ions. The difference between the metal ion adsorption capacities was attributed to the different number of chelating groups of the metals. Cu(II) prefers a 1:1 chelate complex, while Pb (II) prefers to form a complex with 2 amidoxime groups. In another study [59], they modified the surface of electrospun PAN nanofibers with hydrazine (NH_2_–NH_2_) for the removal of metal ions from water. They reported adsorption capacities of 114 and 217 mg/g for Cu(II) and Pb (II), respectively. 

**Figure 8:**
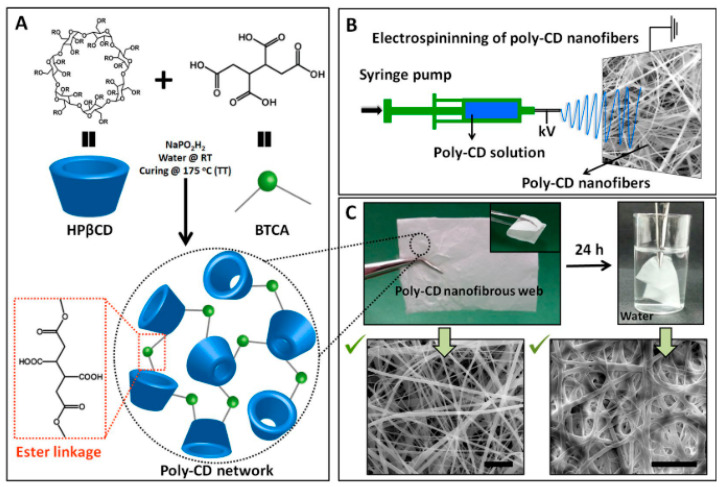
(**A**) Fabrication of a cross-linked poly-CD network structure from HPβCD and BTCA. (**B**) Electrospinning of the CD solutions containing BTCA and an initiator. (**C**) Digital photos of the self-standing and insoluble properties of the poly-CD nanofibrous membranes. The SEM images show poly-CD nanofibers before and after immersing in water for 24 h (scale bar: 10 μm) (Reproduced with permission from [64]. Copyright Springer Nature, 2017.).

Inclusion complexation is another way of capturing chemicals from liquids. The capture mechanism is based on the noncovalent entrapment of a target/guest compound into a chemical compound/host containing cavities. Cyclodextrins (CDs) are well known hosts for the formation of inclusion complexes. CDs are natural cyclic oligosaccharides with a hydrophilic outer surface and a lipophilic central cavity, containing α(1,4)-linked glucopyranose units (Figure 8) [60,61,62]. They have been extensively used for the separation, purification and filtration of a variety of molecules in cosmetic, food, textile, pharmaceutical, agricultural, and chemical industries [63]. CDs are capable of forming inclusion compounds, mainly through noncovalent/hydrophobic host–guest interactions, though other forces such as Van der Waals and dipole–dipole interactions may affect the binding of the guest [63]. CDs have been used with electrospun nanofibers for the removal of organic chemicals from aqueous solutions. However, as these sugar molecules are water soluble, their physical attachments to the nanofiber surface are often unstable and can easily leach out into aqueous media during filtration [63]. As such, these molecules are often attached to the nanofiber surface with chemical treatments.

In the literature, there are many studies on the CD functionalized nanofibrous membranes for liquid filtration applications [64,65]. For example, Celebioglu et al. [63] grafted Beta-cyclodextrin (β-CD) onto electrospun cellulose acetate (CA) nanofibers by using a click reaction. They demonstrated that the β-CD functionalized nanofibers could remove up to 80% of the phenanthrene from its aqueous solution. CDs were also electrospun into nanofibers without using any carrier polymer matrix. The challenge here is to reduce the water solubility of the nanofibers while maintaining the high surface area, and thus the filtration performance of the nanofibrous membranes. In addition, Uyar and coworkers [60] suggested a facile method for the fabrication of insoluble cross-linked poly-CD nanofibers in the absence of any additional polymeric carriers. They first carried out electrospinning on a CD solution containing Hydroxypropyl-β-cyclodextrin (HPβCD), 1,2,3,4-butanetetracarboxylic acid (BTCA) (as a cross-linking agent), and an initiator, then cured the obtained nanofibers at a high temperature to polymerize the CDs and create cross-linked insoluble poly-CD nanofibers (Figure 8). The poly-CD membranes showed a high Methylene Blue (MB) adsorption capacity of 124.1 mg/g at pH 9 and removed up to 90% MB from a 40 mg/L aqueous dye solution under an extremely high flux (3840 L m^−2^ h^−1^). 

### 2.4. Affinity Adsorption 

Affinity membranes can capture targeted molecules based on highly specific binding interactions rather than molecular weight, size, or charge. An affinity adsorbent is made by immobilizing a ligand onto a substrate. The immobilized ligand is then contacted to the solution containing a molecule that binds specifically to that ligand. Electrospun nanofibers are an ideal material for the fabrication of affinity membranes because they can provide a high specific surface area and a highly tortuous porous structure for capturing ligands. In this part, we review the common ligands for the production of electrospun affinity membranes.

**Figure 9:**
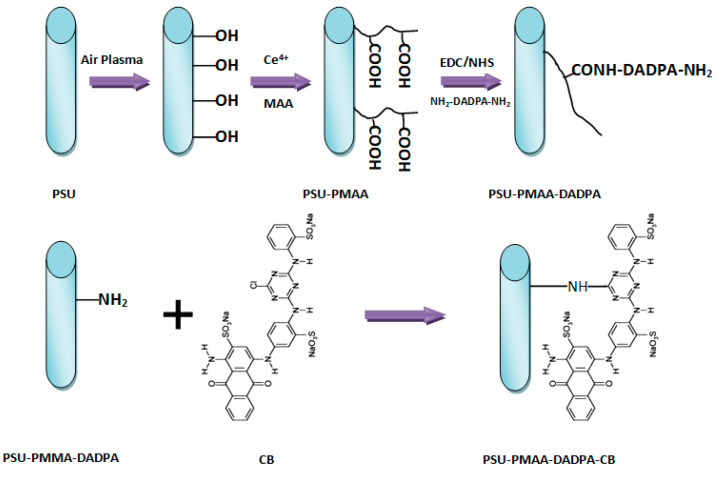
Scheme of the surface modification of electrospun PSU nanofibers with CB.

Cibacron blue F3GA (CB) is an excellent ligand for the purification of many enzymes and blood proteins because its anthraquinone compound (i.e., polycyclic aromatic hydrocarbon (Figure 9)) mimics the structure of protein substrates [66,67]. CB has been covalently reacted with various amino or hydroxyl polymers such as chitosan beads, poly(styrene-co-divinylbenzene) microparticles, polyamides, and cellulose membranes for protein immobilization and separation [68]. Ma et al. [67] covalently attached a CB dye ligand to regenerated cellulose nanofibers and the resultant membranes captured bilirubin pigment and BSA protein with low adsorption capacities of 4 and 13 mg/g, respectively. In other studies [66,69], they functionalized the surface of polyethersulfune (PES) and polysulfune (PSU) nanofibers with CB dye through a series of surface modification steps on the membrane (Figure 9). The capture capacity of the membrane towards BSA protein was low because of a high membrane porosity and a low specific surface area. Furthermore, Zhang et al. [68] enhanced the surface chemistry of electrospun chitosan/nylon-6 nanofibers with CB and reported a papain adsorption capacity of 70 mg/g for the membrane. In another study, they produced electrospun chitosan nanofibers attached to Cibacron Blue F3GA (CB) and the obtained membranes adsorbed bromelain enzyme with a capture capacity of 161.6 mg/g. Such high adsorption was attributed to a very large specific surface area and the superior mechanical properties of the membranes [70].

**Figure 10:**
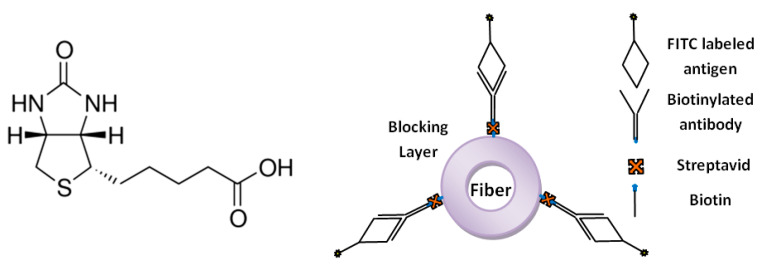
Chemical structure of biotin (left) and scheme of antibody/antigen immobilization on the fiber surface with the biotin/streptavidin capture mechanism (right). One active site of avidin is occupied by the biotin and the three vacant sites remain for further binding with biotinylated rabbit anti-goat IgG. The antigen–FITC is finally attached to the nanofiber surface by antibody–antigen interactions.

**Figure 11:**
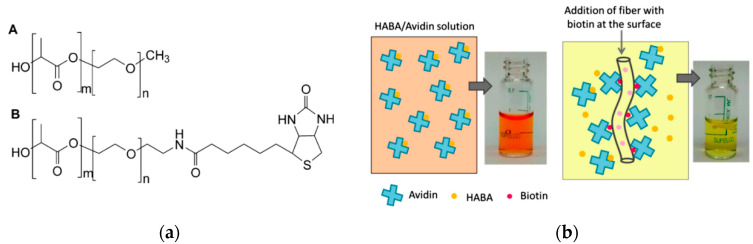
Molecular structures of PLA-b-PEG and PLA-b-PEG-Biotin copolymers (**a**), illustration of PLA/PLA-b-PEG/Biotin nanofibers for the removal of avidin from a 4′-hydroxyazobenzene-2-carboxylic acid (HABA)/Avidin solution (**b**). Initially, avidin and HABA form a complex with an orange color. When nanofibers containing biotin are added to the solution, avidin binds biotin, breaking the HABA/avidin complex and leading to a color change. (Reproduced with permission from [74]. Copyright American Chemical Society, 2017).

Biotin, or vitamin B, is another ligand for the production of affinity nanofibrous membranes. The high affinity of biotin to proteins such as avidin and streptavidin has been utilized in many protein and nucleic acid purification and detection methods. These proteins can bind up to four biotin molecules, which are normally conjugated with enzymes, bioreagents, and proteins (Figure 10). The bond formation is very rapid and, once formed, is not disturbed by the extremes of pH, temperature, organic solvents, or other denaturing agents [71,72]. Our research group previously produced biotin affinity nanofibers for the immobilization of biomolecules [28,73]. Gonzalez et al. [69] incorporated biotin into electrospun nanofibers with poly(lactic acid)-block-poly(ethylene glycol) (PLA-b-PEG) block copolymers. The PLA-b-PEG provided hydrophilicity and aided the migration of biotin to the nanofiber surface (Figure 11). The addition of PLA–PEG to the membrane considerably increased the amount of available biotin on the fiber surface by up to 60% for avidin adsorption. In a follow-up study [74], they also tailored the block length of PLA–PEG and covalently attached the biotin onto the copolymer (Figure 11). They reported the same relative amount of biotin on the nanofiber surface by adding 85% less biotin compared to the former study.

Proteins A and G are other affinity ligands capable of specifically reacting with the antibody IgG. Protein A is cell wall protein component—a single polypeptide chain—containing four binding sites that specifically capture the Fc region of IgG. Protein G is a bacterial cell wall protein containing two high affinity IgG binding sites as well as albumin binding domains. Recently, protein A/G has been developed by combining four Fc-binding domains from protein A with two domains from protein G with the albumin binding site of protein G eliminated. Compared to A and G, Protein A/G has a stronger affinity effect, lower nonspecific binding, and higher stability in a broader range of pH values [44,69,75,76]. Ma et al. [69] covalently bonded protein A/G on the surface of electrospun polyethersulfone (PES) nanofibers via EDC/NHS coupling agents. They reported that the membranes can selectively adsorb IgG from a IgG/BSA protein mixture. However, the obtained IgG capture capacity was low (4.5 mg/mL) due to the relatively small specific surface area (4 m^2^/g), a high membrane porosity of 71%, and a wide pore size distribution. Moreover, Zhu and Sun [40] applied various surface activating agents to PVA-Co-PE nanofibrous membranes to generate reactive sites for protein ligand immobilization. Glutaraldehyde (GA) provided the highest antibody binding capacity thanks to the potential space effect of the oligomer units formed by GA molecules. They reported a high A/G adsorption capacity of 77.2 mg/g and a high IgG binding capacity of 61.4 mg/g for the GA pre-activated PVA-co-PE nanofibers. 

### 2.5. Magnetic Adsorption

**Figure 12:**
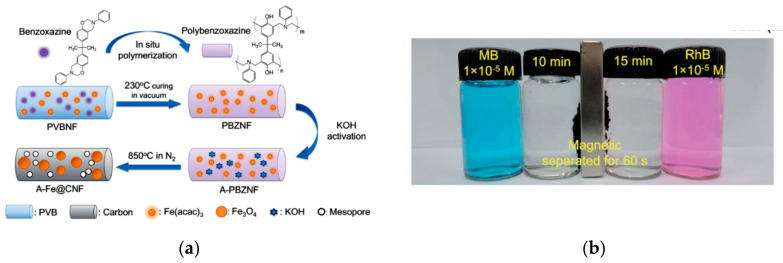
Scheme of the in situ polymerization method for the synthesis process of A-Fe@CNFs (**a**), image of the nanofibers after adsorption of MB (10 min) and RhB (15 min) (**b**) (Reproduced with permission from [78]. Copyright The Royal Society of Chemistry, 2012).

Magnetic nanomaterials have recently attracted interest for the separation of biomolecules and catalysts. In particular, one dimensional (1D) magnetic carbon nanofibers have attracted great interest because of their large surface area, low density, good chemical resistance, and excellent charge transport capability [77]. The principle capture mechanism is based on the magnetic adsorption between metals incorporated inside nanofibers and an external magnetic field. Several researchers used the magnetic mechanism in nanofibers for the separation of species. Si et al. [78] fabricated Fe_3_O_4_ carbon nanofibers based on 1D polybenzoxazine (PBZ) by using an in situ polymerization method. The complete adsorption of Methylene Blue (MB) and Rhodamine B (RhB) dyes was obtained in 10 and 15 min, respectively, due to the extremely high surface area (1885 m^2^/g) and high porosity (2.325 cm^3^/g) of the nanofibers (Figure 12). Moreover, the dye adsorbed on the nanofibers was easily separated from the solution by an external magnet. In another study, Hong et al. [79] anchored NiFeO_4_ nanoparticles on electrospun silica nanofibers by a dip-coating method. The obtained flexible magnetic nanofibers could effectively separate organic dyes from water due to their high porosity, large surface area, and excellent magnetic responses.

**Figure 13:**
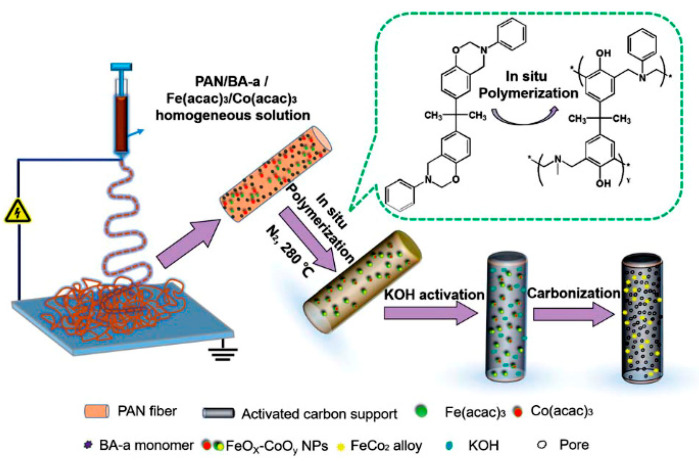
Scheme of FeCO_2_ crystals supported on nitrogen-doped porous carbon fibers (Reproduced with permission from [81]. Copyright The Royal Society of Chemistry, 2017).

In addition, Han et al. [77,80] fabricated Fe_3_O_4_/carbon nanocables with core–shell structures using coaxial electrospinning. The Fe_3_O_4_ was embedded in the core of the carbon matrix to provide the nanocables with electrical and magnetic properties. The nanocables with a specific surface area of 322.6 m^2^/g and a pore size of 33.6 nm demonstrated excellent magnetic separation performance and effectively captured Rhodamine B and Cu^2+^ ions from an aqueous solution. Additionally, Lue et al. [78] fabricated ferro-cobalt (Fe-Co) crystal-doped carbon fibers via combing electrospinning, activation, and carbonization processes for the removal of organic pollutants in water (Figure 13). The polyacrylonitrile/polybenzoxazine (PAN/PBZ) nanofibers were first produced with co-electrospinning and an in situ doping method. Then, the nanofibers were activated with KOH and carbonized at a high temperature to create porous magnetic FeCo carbon fibers. They demonstrated that the nanofibers can completely remove bisphenol-S, chlorophenol, sulfa- methoxazole, and phenol in 30, 30, 15, and 25 min, respectively. 

## 3. Future Outlook

Electrospun nanofibers have been proven to be an excellent material for the fabrication of membranes for chemical separation. These nanofibers can provide large surface area to volume ratios, tunable surface morphology, tortuous porous structure, low mass transfer limitation, and multiple sites for attachment or interaction (Table 1). Despite the recent development of such nanofibers for membrane separation, there are still many challenges that need to be considered. Most of the work has focused on the separation of only one species (e.g., protein, dye, metal) from a solution. The design and synthesis of polymer nanofibers capable of the concurrent separation of two or more chemicals with similar and different structures is a substantial challenge. Additionally, the reusability of the nanofibrous membranes is still a vital issue. While for ion exchange membranes the adsorbed nanofibers can be easily reused by mildly eluting/rinsing the membrane with appropriate aqueous acidic/basic solutions, for other membranes (e.g., covalent and affinity), because of a strong interaction between the nanofibers and the target molecule, multi-step chemical reactions are often required, which may significantly damage the nanofibers’ shape and performance. Developing better methods for reusing such membranes can increase operational flexibility and reduce material and labor costs. Another challenge is related to the scalability of the nanofiber membranes. Most of the synthesis/treatment methods, such as plasma treatment and in situ polymerization, are limited to the laboratory and not appropriate for industrial scales. Moreover, the poor strength and toughness of electrospun nanofibers are important issues and further attention must be given to improving the membranes’ mechanical/dimensional stability. These challenges would require more research and understanding of various adsorption processes and the structural properties of nanofiber membranes.

## Figures and Tables

**Table 1:** Summary of electrospun nanofibrous membranes for chemical separation.

Capture Mechanism	Nanofiber Materials	Adsorption Capacity	Ref.
Ion exchange	WK/SF	2.88 μg/mg (Cu(II))	[17]
	Chitosan	600 (Cu(II))) and 400 (Ag(I)) mg/g	[18]
	PVA/MAH	177 mg/g (LYZ)	[19]
	EVOH–CCA	284 mg/g (LYZ)	[20]
	PDA/PEI@PVA/PEI	1180 (Ponceau S), 1290 mg/g (MB)	[21]
	Silk-PMDA	710 mg/g (LYZ)	[22]
	PAN/PET–Cellulose Nanowhiskers	68 mg/g (CV)	[24]
	SiO_2_@CNF	30 mg/g (BSA)	[25]
	PVA/PMA	476.53 mg/g (LYZ)	[31]
	PS/PEI	1000 (Sunset Yellow FCF), 357.14 mg/g (Cd(II))	[32]
	PAN–COOH	105 mg/g (LYZ)	[33]
	CNF–COOH	200 mg/g (LYZ)	[34]
	PAN–LYS	425.49 mg/g (pepsin) and 54.98 mg/g (LYZ)	[35]
	(Zr6O4(OH)_4_(COOH)_6_(BTC)_2_	276.96 mg/g (Hg)	[38]
	Fe_3_O_4_–(H_2_O)_2_(BTC)_2_·*n*H_2_O	299.66 mg/g (Hg)	[38]
Covalent attachment	Silk fibroin	56.6 μg/mg (CT for 205 nm fiber diameter)	[47]
	Collagen-modified PANCAA	9.15 mg/g (Lipase)	[50]
	PSF	0.8 mg/g (Lipase)	[51]
chelation	Chitosan	485.44 (Cu(II)) and and 263.15 mg/g (Pb(II))	[54]
	PAA/PVA	0.142 mmol/g ((Cu(II))	[55]
	PVA cross-linked PEI	70.92 (Cu(II)), 121.95 (Cd(II)) and 94.34 mg/g (Pb(II))	[56]
	PVA/SiO_2_–SH	489.12 mg/g (Cu(II))	[57]
	(NaOH Hydrolyzed PAN) H-ePAN	31.3 mg/g (Cu(II))	[58]
	PAN-oxime	52.70 (Cu(II)) and 263.45 mg/g (Pb (II))	[53]
	Hydrazine-modified PAN	114 (Cu(II)) and 217 mg/g (Pb (II))	[59]
	Poly-cyclodextrin	124.1 mg/g	[64]
affinity	Cellulose-attached CB	4 (bilirubin) and 13 mg/g (BSA)	[67]
	Chitosan/nylon-6-attached CB	70 mg/g (Papain)	[68]
	Chitosan-attached CB	161.6 mg/g (Bromelain)	[70]
	(PLA-b-PEG)/Biotin	107.2 mg/g (avidin)	[73]
	PES-attached protein A/G	4.5 mg/mL (IgG)	[69]
	PVA-Co-PE-attached protein A/G	61.4 mg/g (IgG)	[44]
Magnetic adsorption	A-Fe@CNFs	Complete adsorption of MB and RhB dyes	[78]
	PAN/PBZ	Complete removal of bisphenol-S, chlorophenol, and phenol	[81]
